# Role of TLR5 in the Translocation and Dissemination of Commensal Bacteria in the Intestine after Traumatic Hemorrhagic Shock

**DOI:** 10.1155/2021/6417658

**Published:** 2021-11-19

**Authors:** Yun Zhang, Jian Zhang, Tao Xu, Cheng Zhang, Wen-Qiao Yu, Tao Wei, Bo Zhang, Qi Chen, Jun-Yu Qiu, Hai-Jun Li, Ting-Bo Liang

**Affiliations:** ^1^Department of Hepatobiliary and Pancreatic Surgery, The First Affiliated Hospital, Zhejiang University School of Medicine, Hangzhou, China; ^2^Zhejiang Provincial Key Laboratory of Pancreatic Disease, Hangzhou, China; ^3^Innovation Center for the Study of Pancreatic Diseases, Hangzhou, China; ^4^Department of Surgical Intensive Care Unit, The First Affiliated Hospital, Zhejiang University School of Medicine, Hangzhou, China; ^5^Department of General Surgery, Shenzhen Luohu People's Hospital, The Third Affiliated Hospital of Shenzhen University, Shenzhen, China

## Abstract

Enterogenous infection is a major cause of death during traumatic hemorrhagic shock (THS). It has been reported that Toll-like receptor 5 (TLR5) plays an integral role in regulating mucosal immunity and intestinal homeostasis of the microbiota. However, the roles played by TLR5 on intestinal barrier maintenance and commensal bacterial translocation post-THS are poorly understood. In this research, we established THS models in wild-type (WT) and *Tlr5^−/−^* (genetically deficient in TLR5 expression) mice. We found that THS promoted bacterial translocation, while TLR5 deficiency played a protective role in preventing commensal bacteria dissemination after THS. Furthermore, intestinal microbiota analysis uncovered that TLR5 deficiency enhanced the mucosal biological barrier by decreasing RegIII*γ*-mediated bactericidal activity against G^+^ anaerobic bacteria. We then sorted small intestinal TLR5^+^ lamina propria dendritic cells (LPDCs) and analyzed T_H_1 differentiation in the intestinal lamina propria and a coculture system consisting of LPDCs and naïve T cells. Although TLR5 deficiency attenuated the regulation of T_H_1 polarization by LPDCs, it conferred stability to the cells during THS. Moreover, retinoic acid (RA) released from TLR5^+^ LPDCs could play a key role in modulating T_H_1 polarization. We also found that gavage administration of RA alleviated bacterial translocation in THS-treated WT mice. In summary, we documented that TLR5 signaling plays a pivotal role in regulating RegIII*γ*-induced killing of G^+^ anaerobic bacteria, and LPDCs mediated T_H_1 differentiation via RA. These processes prevent intestinal bacterial translocation and enterogenous infection after THS, suggesting that therapeutically targeting LPDCs or gut microbiota can interfere with bacterial translocation after THS.

## 1. Introduction

Traumatic hemorrhagic shock (THS) is highly prevalent and is one of the leading contributors to morbidity and mortality worldwide. The direct effects of traumatic injuries, secondary hemorrhage, and shock are usually severe; however, increasing evidence has revealed that the major cause of death after THS is severe systemic infection [1]. Moreover, several studies have shown that infections secondary to THS mostly result from intestinal bacterial translocation [2, 3]. However, the details of the underlying mechanisms are not well understood, given that the immunological and biological barriers have not been fully explored. These barriers are widely acknowledged to work with the mucosal mechanical barrier in the intestine.

The gut biological barrier is primarily composed of commensal anaerobic bacteria. It consists of a complex community of more than 1000 different species of commensal eukaryotes, archaea, and bacteria [3, 4]. Numerous anaerobes and other commensal bacteria reportedly serve as a critical biological barrier that blocks contact between pathogens and the intestinal epithelium [5]. Accordingly, disrupting the gut microbial communities of the host using antibiotics may worsen clinical outcomes [6]. However, the mechanisms involved remain largely unexplored, warranting further studies.

The intestinal immune system is another major component of the gut barrier. The system is based entirely on secretory IgA-mediated neutralization, lymphocyte activation beneath the intestinal mucosa, and the bactericidal actions of effector cells. Among these cells, the lamina propria dendritic cells (LPDCs) [7–11] play a direct and critical role in modulating the gut mucosa-related immunological state. LPDCs are extensively localized beneath the intestinal epithelium and have much easier access to enteric bacteria. Moreover, LPDC maturation and their ability to exert cellular immune functions may be highly affected by hypoxia after THS [12].

The function of the intestinal mechanical barrier largely depends on the integrity of the mucus layer, epithelial layer, and tight junction. Furthermore, accumulating evidence indicates that, in addition to constituting the intestinal mechanical barrier, intestinal epithelial cells (IECs) also greatly impact both the local mucosal immunological state and the gut commensal microbiota [13, 14].

Commensal microbiota, mucosal immunity, and the intestinal mechanical barrier work together to maintain a healthy microbial composition and intestinal homeostasis. Furthermore, the gut barrier, composed of the above three factors, prevents invasion by pathogenic species. How do these three factors interact and cooperate with each other in local circumstance?

Under homeostatic conditions, there is constant crosstalk between immune cells and IECs via a complex cytokine network, and cytokines contribute greatly to intestinal homeostasis by regulating key cellular processes such as cell death, proliferation, molecular transport, and inflammatory responses against pathogens, including the IL-1 family, like IL-1*β* and IL-18 [15]. Another important cytokine, IL-22, has been documented to play a key role in regulating mucosal homeostasis by promoting IEC survival during bacterial infection by secretion of antimicrobial peptides like RegIII*β* and RegIII*γ* [16]. Vice versa, intestinal microbiota also influences cytokine production by interacting with IECs or immune cells via Toll-like receptors (TLRs) [17].

TLRs are pattern-recognition receptors that sense pathogens and then produce various immunoregulatory factors. Toll-like receptor 5 (TLR5) is highly expressed in the two main cellular compartments of the intestinal mucosa: the IECs and DCs. In both cases, the receptor recognizes bacterial flagellin and induces downstream inflammatory responses [7, 18]. Studies have demonstrated that TLR5 expressed by professional immune cells strongly influences host mucosal immunity [9, 19]. Host immunity, in turn, substantially influences host-microbiota homeostasis [11, 20]. It has been reported that TLR5^+^ LPDCs that are stimulated by flagellin produce retinoic acid (RA), which induces the differentiation of antigen-specific, type II interferon (IFN*γ*)-producing T helper cells (T_H_1 cells) [8]. Importantly, T_H_1 cells inhibit intestinal bacterial translocation [21, 22], while IFN*γ* is mostly associated with providing protection against a broad range of intracellular microorganisms [23]. Furthermore, it has been reported that systemic administration of flagellin or LPS induced RegIII*γ* expression in intestinal epithelial and paneth cells, which could kill G^+^ bacteria [13, 20]. Indeed, the gut microbiota is shaped both by the environment and host genetics. In this regard, the innate immune system has long been known to contribute to defending the host against infection by pathogenic microbes and has now been suggested to play a key role in regulating the gut microbiota. Notwithstanding that TLR5 plays an important role in mucosal immunity and microbiota homeostasis, its influence on post-THS intestinal barrier maintenance, commensal bacterial translocation, and systemic infection is largely unknown.

Here, we provided evidence that post-THS infection is caused by the translocation and dissemination of certain strains of bacteria that originate from the host gut, and TLR5 has direct and significant effects on the composition of the gut microbial community and on mucosal immunity.

## 2. Materials and Methods

### 2.1. Mice

*Tlr5^−/−^* (genetically deficient in TLR5 expression, C57BL/6 background) mice were purchased from the Jackson Laboratory (Stock No: 008377). OT-II mice were a kind gift from Professor Linrong Lu (Institute of Immunology, Zhejiang University). C57BL/6 mice (aged 8–12 weeks and weighing 20–25 g) were purchased from the Animal Resource Center of Zhejiang University, School of Medicine. We crossed heterozygous *Tlr5^+/−^* mice derived from *Tlr5^+/+^* (wild type, WT) with *Tlr5^−/−^* parents. The resulting offspring consisting of *Tlr5^−/−^* and WT neonates were housed together for the following experiments to decrease the possibility of environmental factors influencing the microbial composition of gut communities. All animals were housed in clean vivaria and fed standard mouse chow-based diets. The study protocols were approved by the *Institutional Laboratory Review Board* and were in accordance with the principles stated in the *Guide for the Care and Use of Laboratory Animals-Eighth Edition* (National Institutes of Health publication, 2011).

### 2.2. THS Model Establishment

An experimental mouse model of THS was established as described previously with some modifications [24]. Briefly, the mice were anesthetized through intraperitoneal administration of phenobarbital sodium (100 mg/kg). A small incision was made in the right neck skin; then, the right jugular vein and right carotid artery were catheterized for fluid resuscitation and bleeding, respectively. Trauma was induced by fracturing the left femur in an open, middiaphyseal transverse fashion. Subsequently, mice were bled to obtain a mean arterial blood pressure (MAP) of 30 ± 5 mmHg for 90 minutes, followed by resuscitation for 20 minutes with lactated ringer's solution at a constant rate to control shock. Finally, the catheters were removed, the blood vessels were ligated, and the incisions were closed. A sham group of mice underwent the same anesthetic and vessel catheterizing procedures and fluid resuscitation but without THS. Adequate measures were taken to minimize the suffering of animals.

### 2.3. Bacterial Translocation Evaluation

Bacterial translocation was assessed using a bioluminescent derivative of *Citrobacter rodentium*, *ICC180* (*C. rodentium*, a kind gift from Professor Gad Frankel, Imperial College London) as previously described [25, 26]. The strain was incubated at 37°C in LB medium supplemented with kanamycin (50 *μ*g/ml). Forty-eight hours after THS or sham operations, mice were inoculated by oral gavage with 200 *μ*l of a *C. rodentium* suspension in PBS at a density of 2.5 × 10^7^ colony forming units (CFUs)/*μ*l. The whole animal was imaged one hour after the bacterial challenge using a spectrum optical in vivo imaging system (IVIS) (Kodak, Rochester, USA). The presence of *C. rodentium* was measured according to our previous description [27]. The animals were sacrificed by cervical dislocation approximately 5 hours after imaging. Sample homogenates obtained from mesenteric lymph nodes (MLNs), livers, spleens, and blood were plated on LB agar culture plates and grown under aerobic conditions at 37°C for 12 hours. Then, the bacterial CFUs were counted.

### 2.4. Microbiome Analysis

Pyrosequencing of the 16S rRNA gene was performed by ProMe Gene (Shenzhen, China). Fecal DNA was isolated and purified by standard phenol-chloroform methods [28]. Samples were sequenced by MiSeq sequencing (Illumina MiSeq), as previously described [29]. Data were analyzed using the QIIME 1.8.0 software package (http://qiime.org/) [30].

### 2.5. Immunohistochemistry

Tissue slides were incubated with the corresponding primary RegIII*γ* antibody (Abcam, Cambridge, England) or IFN*γ* antibody (Abcam) and then incubated with HRP-conjugated antibody against rabbit or mouse IgG using a Histostain-Plus Kit (ZSGB-BIO, Beijing, China). The slides were counterstained with hematoxylin and finally inspected under a microscope (Leica, Wetzlar, Germany).

### 2.6. Determination of MAP and Microvascular Tissue Perfusion in the Intestinal Lamina Muscularis

The mean arterial pressure (MAP) of the mouse was measured with a tail-cuff system (BP-98A; Softron, Tokyo, Japan), as previously described [31, 32]. A laser Doppler imaging system (Moor Instruments, Axminister, England) was used to determine the microvascular tissue blood perfusion of the intestine [27].

### 2.7. Determination of Transepithelial Electrical Resistance (TEER) and Mucosal Permeability of the Small Intestine

The ex vivo permeability of the intestinal mechanical barrier was assessed by measuring the TEER in intestinal segments (2–3 cm) from the distal ileum mounted in an Ussing chamber (Physiologic Instruments, San Diego, USA), as previously described [27]. The TEER data were analyzed using Analyze & Acquire Revision II software (Physiologic Instruments). The in vivo permeability of the intestine was measured with undigested FITC labeled dextran (4 kDa, Sigma-Aldrich, St. Louis, USA) as previously described [27].

### 2.8. Fluorescence-Activated Cell Sorting (FACS) and Flow Cytometric (FCM) Analysis

LPDCs were isolated from the small intestine as CD11c^+^ MHCII^+^ cells or CD103^+^ CD8a^−^ cells, as previously described [8, 33].

All antibodies were purchased from BD Biosciences (San Jose, USA). After blocking the Fc receptors, DC subsets were sorted based on the expression of CD11c (HL3) plus MHCII (M5/114) or CD103 (M290) plus CD8a (53-6.7) by using the FACS Aria II system (BD Biosciences). We routinely obtained purity of over 95% for the sorted LPDCs. TLR5 expression on the LPDCs was evaluated with quantitative real-time PCR and western blotting. Single-cell suspensions from the spleens and thymuses of OT-II transgenic mice were prepared using a mechanical trituration method followed by Ficoll density-gradient centrifugation. Naïve CD4^+^ T cells were sorted according to the positive expressions of CD4 and CD62L and negative expressions of CD8 and CD44. We routinely obtained purity of over 95% for the sorted naïve CD4^+^ T cells.

FCM analysis was performed using a Canto-II system (BD Biosciences). After blocking the Fc receptors, cells were incubated with CD45 (30-F11), CD4 (GK1.5), IFN*γ* (XMG1.2), CD11c (HL3), MHCII (M5/114), CD80 (16-10A1), and CD86 (GL-1), according to the detailed staining panels of T helper (T_H_) cell subgroups and LPDCs. Prior to staining, T cells were stimulated at 37°C for 5 hours with a cell activation cocktail (consisting of BFA (3 *μ*g/ml), monensin (1.4 *μ*g/ml), PMA (50 ng/ml), and ionomycin (1 *μ*g/ml), MultiSciences, Shanghai, China).

### 2.9. Ex Vivo T Cell Differentiation

OT-II transgenic naïve CD4^+^ T cells (2 × 10^5^) were cocultured with CD11c^+^ MHCII^+^ LPDCs (4 × 10^4^) in the presence of OVA323-339 (100 *μ*g/ml, InvivoGen, San Diego, USA), without flagellin, with flagellin (1 *μ*g/ml, InvivoGen) only, or with flagellin plus LE540 (1 *μ*g/ml, Wako, Tokyo, Japan). Cells were cultured for four days followed by FCM analysis or sorted for western blotting.

### 2.10. RA Synthesis in LPDCs

The level of retinal aldehyde dehydrogenase (RALDH) reflects the ability of LPDCs to synthesize RA. Herein, we determined RALDH levels using the ALDEFLUOR™ kit (StemCell, Vancouver, Canada) according to the manufacturer's instructions. Cells were resuspended in 0.5 ml of ALDEFLUOR buffer and stained with FITC-conjugated antibodies against CD11c (HL3) and MHCII (M5/114). RALDH expression in CD11c^+^ MHCII^+^ LPDCs was examined by FCM.

### 2.11. Enteric RA Supplementation

RA (Sigma-Aldrich) was resuspended in 0.5% hydroxypropyl methyl cellulose (HarveyBio, Beijing, China) and administered orally at a dosage of 25 *μ*g/g bodyweight. RA gavage was performed 1 day and 1 hour before THS and 1 hour before bacterial gavage as previously described [34].

### 2.12. Quantitative Real-Time PCR

TLR5 expression on LPDCs was evaluated by quantitative real-time PCR, as previously described [35]. Primers for *Tlr5* (italic lower case refers to the gene) and *gapdh* were designed and purchased from Takara (Shiga, Japan). *Tlr5* mRNA expression was normalized against *gapdh*, and the calculations followed the comparative 2^−*Δ*Ct^ method. The sequences of the primers used to amplify *Tlr5* and *gapdh* are as follows: *Tlr5* forward, 5′-GGTGTGATCTTCATGGCCAGCCC-3′; *Tlr5* reverse, 5′-CGTCGCTTAAGGAATTCAGTTCCCG-3′; *gapdh* forward, 5′-AATGGATTTGGACGCATTGGT-3′; and *gapdh* reverse, 5′-TTTGCACTGGTACGTGTTGAT-3′.

### 2.13. Western Blotting

Western blotting was performed as previously described [35]. Anti-TLR5, anti-IFN*γ*, or anti-GAPDH (Abcam) were used as primary antibodies.

### 2.14. Enzyme-Linked Immunosorbent Assay (ELISA)

RegIII*γ* concentrations from the small intestinal luminal wash fluid were determined through ELISA (USCN, Wuhan, China) according to the manufacturer's instructions.

### 2.15. Animal Survival

Mouse survival was monitored for two months every 2 or 3 days after treatments and compared between the following groups: WT and *Tlr5^−/−^* mice, RA-treated and untreated mice, THS mice treated with *C. rodentium* oral gavage, sham-operated mice, sham-operated and *C. rodentium* gavage-treated mice, and THS only mice.

### 2.16. Statistical Analysis

Data were analyzed using GraphPad Prism 7 (San Diego, USA) and expressed as the mean ± standard deviation (SD) or median with interquartile range for box plots as detailed in the figure legends. A nonparametric Kruskal-Wallis test was used for the intergroup comparisons. Repeated measures ANOVA was used to determine the group and time effects for longitudinally monitored variables. One-way ANOVA with Tukey's multiple comparisons test was used to compare multiple groups. Survival analysis was conducted using the Kaplan-Meier method with log-rank tests. A *p* value of <0.05 was considered statistically significant.

## 3. Results

### 3.1. Post-THS Infection Originated from the Host Gut and TLR5 Deficiency Exerted a Protective Effect against the Translocation and Dissemination of Commensal Bacteria

**Figure 1:**
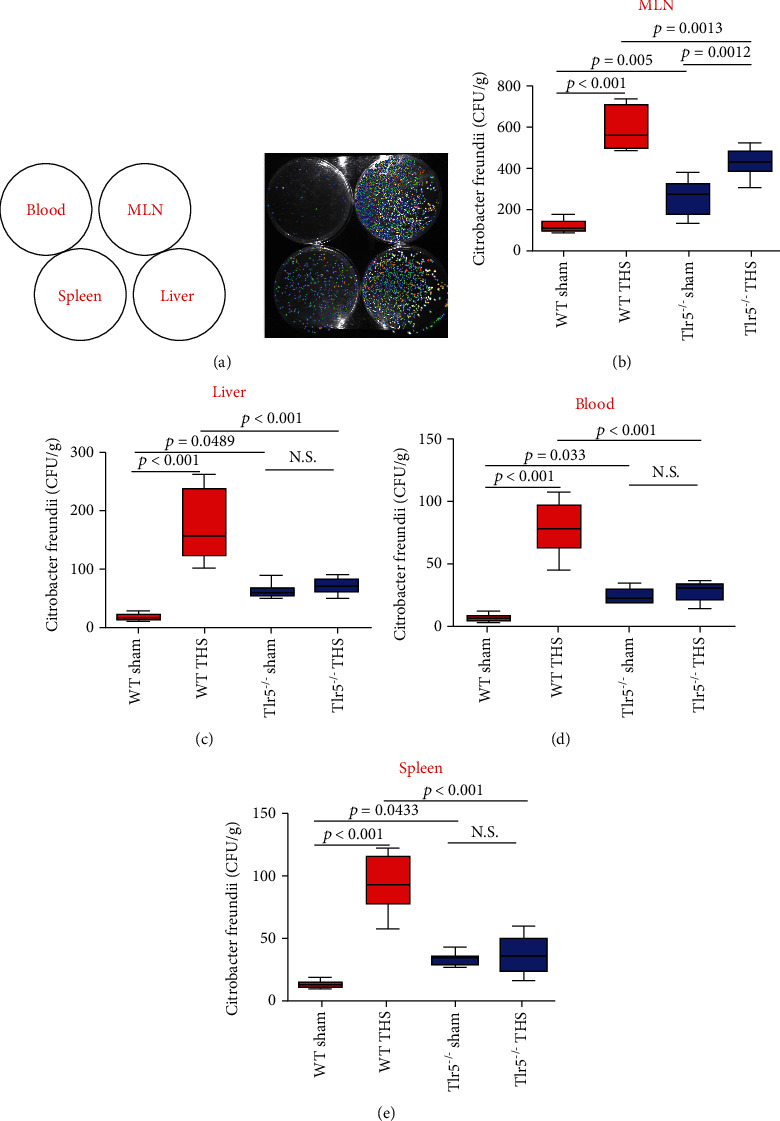
Effect of TLR5 deficiency on the translocation and dissemination of commensal bacteria. Bioluminescent *C. rodentium*, visualized using an in vivo imaging system (IVIS), was used to monitor bacterial translocation. (a) The panel of LB agar plates with different tissue homogenates was shown in the top left corner. Sham-operated mice or mice 48 hours after THS were inoculated by oral gavage with *C. rodentium* and sacrificed approximately 5 hours after the challenge. After that, sample homogenates obtained from the MLNs (b), liver (c), blood (d), and spleen (e) were plated on LB agar culture plates and grown under aerobic condition at 37°C for 12 hours. Subsequently, bacterial CFUs were visualized (a) using IVIS, and bacterial abundance was calculated (b–e). Boxes represent the interquartile range (bottom, 25^th^ percentile; top, 75^th^ percentile), and the line inside represents the median. Whiskers denote the lowest and highest values within 1.5x the interquartile range. Each group contained 8 animals. N.S. indicates not significant. All significant *p* values are marked, and *p* < 0.05 is considered significant by one-way ANOVA test. Data are representative of three independent experiments.

It has been reported that most clinical cases of infection in THS patients involve bacteria commonly known to be commensal and residing in the human intestinal tracts. In the present study, we used the most widely accepted bacterial translocation model that involves *C. rodentium*, a natural murine intestinal pathogen that shares a core set of virulence factors with related human pathogens: enteropathogenic *Escherichia coli* and enterohemorrhagic *Escherichia coli*. Herein, we used bioluminescent *C. rodentium* to accurately and effectively monitor bacterial translocation and confirm whether post-THS infections originated from the host gut (Figure S1A). All mice were gavaged with *C. rodentium* (Figure S1B and Figure 1(a)), and we found that in the sham-operated group, intestinal bacterial translocation into the MLNs (Figure 1(b)) was higher in the *Tlr5^−/−^* mice than in the WT mice (*p* = 0.005). Moreover, after THS, bacterial translocation into the MLNs was markedly elevated in WT (*p* < 0.001) and *Tlr5^−/−^* (*p* = 0.0012) mice. Remarkably, more bacteria translocated into the MLNs of WT mice after THS compared with corresponding bacterial translocation in *Tlr5^−/−^* mice (*p* = 0.0013).

To confirm the involvement of TLR5 in bacterial translocation into the MLNs, we analyzed bacterial translocation into other organs and found that compared with WT, *Tlr5^−/−^* mice were more prone to bacterial translocation into the extraintestinal organs, namely, liver (Figure 1(c), *p* = 0.0489), portal vein blood (Figure 1(d), *p* = 0.033), and spleen (Figure 1(e), *p* = 0.0433). However, after THS, extraintestinal bacterial translocation into the liver (Figure 1(c), *p* < 0.001), portal vein blood (Figure 1(d), *p* < 0.001), and spleen (Figure 1(e), *p* < 0.001) increased markedly in WT mice but not in *Tlr5^−/−^* mice. Furthermore, after THS, bacterial translocation into visceral organs (liver (Figure 1(c), *p* < 0.001), portal vein blood (Figure 1(d), *p* < 0.001), and spleen (Figure 1(e), *p* < 0.001) was significantly lower in *Tlr5^−/−^* mice than in WT mice, suggesting a protective effect from *Tlr5* knockout.

Collectively, the results described above suggested that THS promotes bacterial translocation and raised the question of why TLR5 deficiency played a protective role against commensal bacterial translocation after THS.

### 3.2. TLR5-Dependent Protective Role of RegIII*γ* against G^+^ Anaerobes Altered the Biological Barrier after THS

#### 3.2.1. Microbiota Analysis

**Figure 2:**
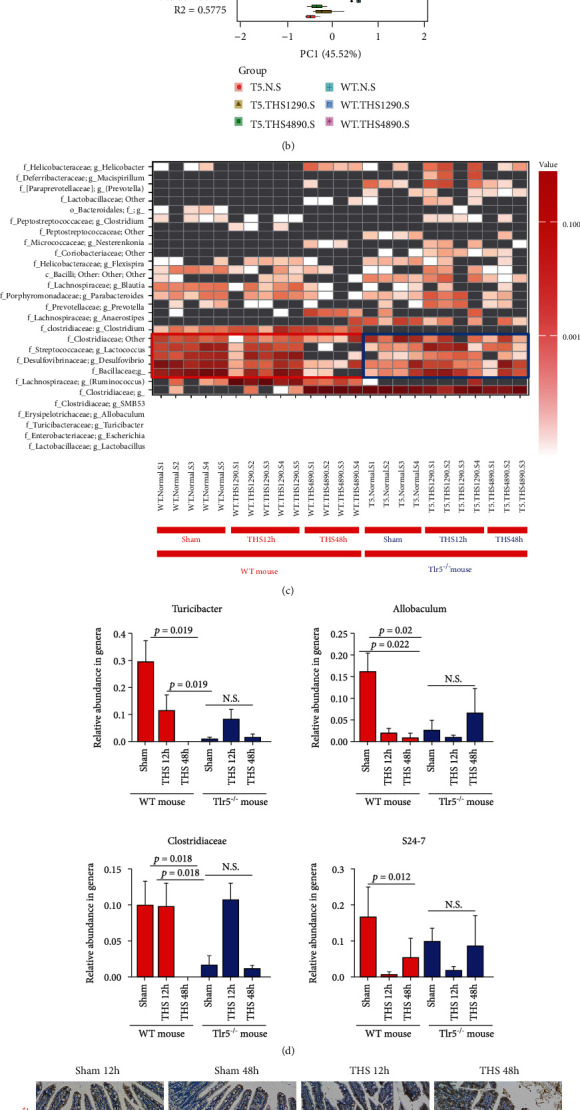
Profile of intestinal mucosal biological barrier after THS. (a) Alpha diversity analysis of microbial communities in the small intestine of sham-operated (WT.N.S (*n* = 5) and T5.N.S (*n* = 4)) mice, 12 hours post-THS (WT.THS1290.S (*n* = 5), T5.THS1290.S (*n* = 4)) mice, and 48 hours post-THS (WT.THS4890.S (*n* = 4), T5.THS4890.S (*n* = 3)) mice. (b) Principal component analysis (PCA) of the above six groups. (c) Heatmap of operational taxonomic units of commensal bacterial genera from sham-operated and post-THS WT or *Tlr5^−/−^* mice. The red box indicates anaerobes in the WT mice, and the blue box indicates anaerobes in the *Tlr5^−/−^* mice. (d) Relative abundance of the bacterial genera or families of microbial communities in the small intestine of sham-operated and post-THS WT or *Tlr5^−/−^* mice. Data represent the mean ± SD. (e, f) Representative images (e) and comparisons (f) of RegIII*γ* staining in the small intestine of sham-operated and post-THS mice of different genotypes were shown. (g) Luminal RegIII*γ* was determined by ELISA. The number of mice in each group is described in (a), except *n* = 8 in €. All significant *p* values are indicated, and *p* < 0.05 is considered significant by one-way ANOVA test. WT indicates wild type, T5 indicates *Tlr5^−/−^*, N indicates sham-operated, S indicates small intestine, 12 h or 48 h indicates the number of hours post-THS, and 90 indicates the shock period of 90 minutes.

To expand the study beyond bioluminescent *C. rodentium*, we studied the total gut microbiota using high-throughput sequencing (amplicon sequencing) of the 16S rRNA gene and relevant bioinformatics tools. We analyzed the microbiota of the small intestine of sham-operated mice (normal) or mice at the 12^th^ and 48^th^ hour of recovery after undergoing 90 minutes of traumatic shock (THS1290 and THS4890, respectively). The data clearly showed a significant increase in ɑ-diversity (especially in the chao1 index, and less striking in the indexes of observed_otus and PD_whole_tree, *p* < 0.05 for all the comparisons indicated in Figure 2(a)) in the *Tlr5^−/−^* mice, suggesting that these mice had a relatively more mature and more stable microbiota structure. Moreover, shifts in the abundance levels and distributions of the existing microbiota were observed. For example, the microbial communities within the small intestines of the sham-operated and the post-THS mice of different genotypes were significantly different from one another, based on principal component analysis (PCA, Figure 2(b), *p* < 0.001). In particular, in addition to the distant UniFrac distance between WT mice and *Tlr5^−/−^* mice, the UniFrac distances among subgroups of *Tlr5^−/−^* mice were shorter than those of WT mice, which meant much higher levels of intersubgroup variations among WT mice than *Tlr5^−/−^* mice. These results suggested that TLR5 deficiency plays an important role in maintaining a relatively stable gut microbiota.

The results obtained from ɑ-diversity and PCA were consistent with results of microbial community analysis of the small intestine, which showed significant differences between WT and *Tlr5^−/−^* mice before and after THS.

As seen in Figures S2A and S2B, the relative abundance of *Firmicutes i*n WT mice (mainly G^+^ anaerobes) decreased 12 hours after THS; however, the difference was not statistically significant. A sharp statistically significant increase in *Firmicutes* was subsequently observed 48 hours after THS (*p* = 0.037), which was mostly accounted for by G^−^ aerobic *Lactobacillus*. In contrast, the abundance levels of *Firmicutes* in the *Tlr5^−/−^* mice did not change significantly over time. Furthermore, the abundance of *Bacteroidetes* (consisting mainly of family *S24.7*, anaerobic) was significantly lower in WT mice 12 hours after THS (*p* = 0.037) and remained low 48 hours after THS. The relative abundance of *Bacteroidetes* was not altered in *Tlr5^−/−^* mice after THS.

At the genus level, most of the significant changes occurred in G^+^ anaerobic populations in the small intestine of WT mice (Figure 2(c) and Figures S2C and S2D), which included *Turicibacter*, *Allobaculum*, *Clostridiales*, *Lachnospiraceae*, *Clostridiaceae*, and *S24.7*. After THS, the abundance levels of most G^+^ anaerobes (Figures 2(c) and 2(d)) decreased sharply. Interestingly, G^−^ aerobic *Escherichia* (Figure S2E) increased significantly 12 hours after THS (*p* = 0.012) and decreased 48 hours (*p* = 0.02) after THS, while G^−^ aerobic *Lactobacillus* (Figure S2F) increased 48 hours after THS (*p* = 0.015 compared with normal, *p* = 0.019 compared with THS1290). Surprisingly, in *Tlr5^−/−^* mice, *Lactobacillus* (Figure 1(c) and Figure S2F) and *S24.7* (Figure 2(d)) were the most dominant genera; however, their levels were not altered after THS. No significant change in the abundance levels of other anaerobes mentioned above was observed.

These findings provided valuable information on the dynamics of microbial populations in the small intestine after THS. By comparing WT and *Tlr5^−/−^* mice, the results demonstrated that TLR5 deficiency plays a pivotal role in maintaining a stable microbiota, especially among anaerobes that participate in building the gut biological barrier. These results also raised questions on why the abundance of anaerobes declined sharply after THS in WT mice.

#### 3.2.2. The Role of RegIII*γ*

RegIII*γ* is a secreted C-type lectin that kills G^+^ bacteria, including the anaerobes mentioned above. Flagellin, a specific agonist of TLR5, can stimulate the expression of RegIII*γ* in intestinal epithelial and paneth cells, which line the entire length of the small intestine. To assess the functional connection between RegIII*γ* and the decline in the abundance of anaerobes, we analyzed the expression profile of RegIII*γ* in the small intestine. As expected, we found low levels of RegIII*γ* expression under normal conditions, regardless of the type of the mice (WT or *Tlr5^−/−^*). However, after THS, intestinal RegIII*γ* production increased considerably, especially in WT mice, although a small decrease was observed at 48 hours (Figures 2(e) and 2(f)), and high levels of luminal RegIII*γ* secreted by the mucosa were sustained (Figure 2(g)).

In short, after THS, RegIII*γ* synthesis increased. This finding, combined with our observation of the severe decline in anaerobes in WT mice, suggested that after THS, the TLR5 pathway is required for RegIII*γ*-mediated killing of G^+^ bacteria, especially G^+^ anaerobes.

### 3.3. TLR5 Did Not Affect the Intestinal Mechanical Barrier after THS

**Figure 3:**
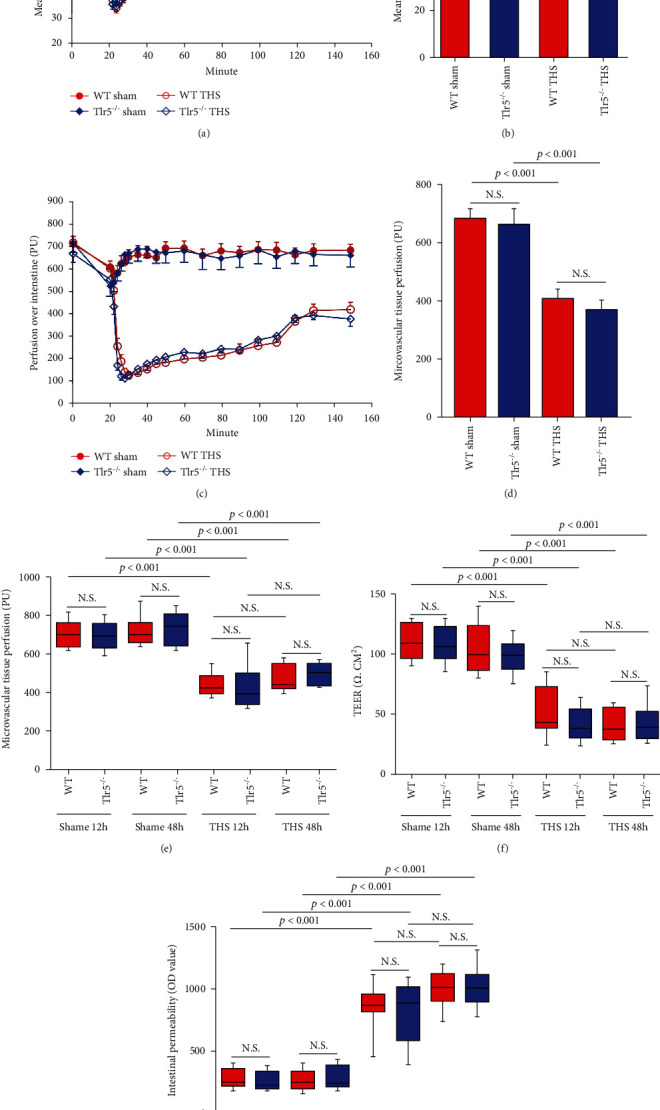
Profile of intestinal mechanical barrier after THS. MAP refers to the mean arterial pressure of the mouse and was monitored during THS and fluid resuscitation (a) and compared with the value obtained at the end of fluid resuscitation (b). Microvascular tissue perfusion was monitored during THS and fluid resuscitation (c) and compared with the value at the end of fluid resuscitation (d) and compared with the values at 12 and 48 hours after THS (e). Ex vivo transepithelial electrical resistance (TEER) (f) and in vivo permeability (g) of the small intestine were measured and compared. Data are expressed as the mean ± SD and represent three independent experiments involving 8 animals per group. All significant *p* values are marked, and *p* < 0.05 is considered significant by the repeated measures ANOVA test for MAP and microvascular tissue perfusion and by the one-way ANOVA test for other comparisons.

The contribution of the mechanical barrier to bacterial translocation after THS was observed from clear decreases in MAP and microvascular tissue perfusion of both WT and *Tlr5^−/−^* mice after THS (Figures 3(a) and 3(c)). MAP recovered to a normal level after fluid resuscitation (Figures 3(a) and 3(b)), although the microvascular tissue perfusion remained low (Figures 3(c) and 3(d)), even 12 and 48 hours after THS (Figure 3(e), *p* < 0.001).

Consistent with our MAP and microvascular tissue perfusion findings, the TEER (Figure 3(f), *p* < 0.001) clearly decreased, and the intestinal permeability (Figure 3(g), *p* < 0.001) increased remarkably in both WT and *Tlr5^−/−^* mice after THS. Intriguingly, no significant difference in all the above parameters was found between the WT and *Tlr5^−/−^* mice (Figures 3(b), 3(d), and 3(g)).

Collectively, the changes in MAP and microvascular tissue perfusion through time showed that, with intestinal microcirculation dysfunction, our model successfully mimicked hypotensive shock commonly observed in clinical practice. Although fluid resuscitation transiently restored MAP levels, perfusion of the intestinal microcirculation remained low. These findings might explain the destruction of the intestinal mechanical barrier manifested by mucosal permeability elevation and TEER decline.

### 3.4. *Tlr5* Knockout Attenuated LPDCs' Ability to Polarize T_H_1 and Also Conferred Stability to the Cells during THS, Which Was Regulated by Retinoic Acid

#### 3.4.1. Identification and Basic Characteristics of TLR5^+^ LPDCs

Injury to the mechanical barrier is a crucial factor after THS [36, 37]. However, the immunological barrier is even more important. TLR5^+^ LPDCs are important APCs and play pivotal roles in regulating cellular immunity in the lamina propria (LP) of the small intestine via RA synthesis [8]. Therefore, we sought to investigate whether T_H_1 cell differentiation was regulated by TLR5^+^ LPDCs in the LP of the small intestine. In a study by Fujimoto et al. [10], identifying LPDCs that specifically express TLR5 was based on CD103 positive and CD8a negative staining. However, when we repeated their experiments, TLR5 expression was also detected in CD103^−^ cells (Figure S3A). Therefore, we instead selected CD11c and MHCII as markers to enable the sorting of specific groups of LPDCs. We first verified that CD11c^+^ MHCII^+^ LPDCs specifically expressed TLR5 (Figure S3B). TLR5 was not expressed in the CD11c^−^ cells sorted from WT mice or in all cells derived from *Tlr5^−/−^* mice (Figure S3B). For convenience, we termed the subgroup of CD11c^+^ MHCII^+^ TLR5^+^ LPDCs as LPDCs.

Moreover, the frequencies of LPDCs (Figures S3C and S3D) and the expression levels of DC maturation markers CD80 (Figure S3E) and CD86 (Figure S3F) in the small intestines of *Tlr5^−/−^* and WT mice were similar. These results suggested that deleting *Tlr5* in DCs does not affect the development or maturation of LPDCs, even after THS.

#### 3.4.2. *Tlr5* Knockout Attenuated the Regulation of T_H_1 Polarization by LPDCs and Also Conferred Stability to the Cells during THS

**Figure 4:**
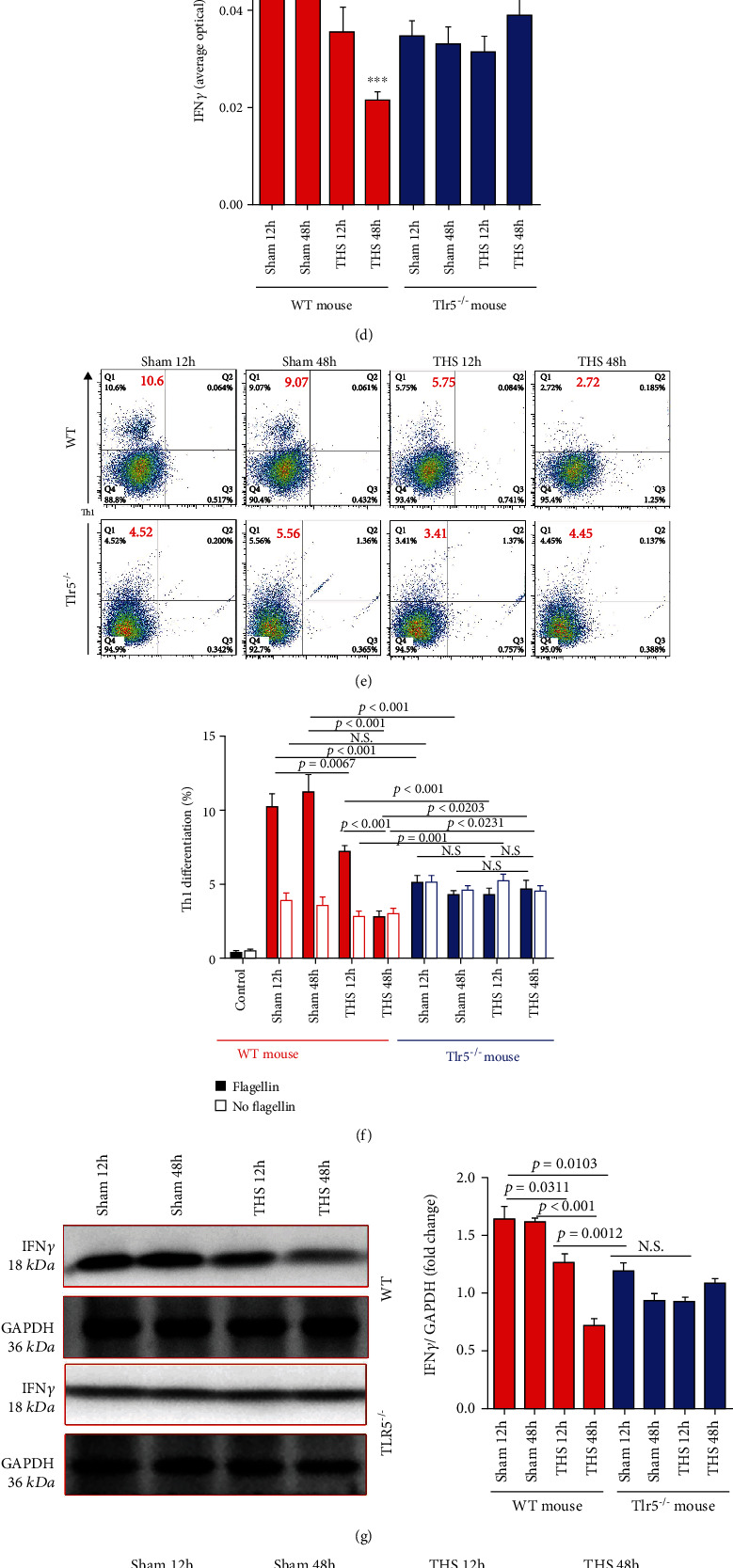
Role of TLR5 in regulating the gut immunological barrier in LPDCs. The ability of LPDCs to induce T_H_1 cell polarization was evaluated in vivo and ex vivo. (a, b) T_H_1 cells in the LP of the small intestine were analyzed (a) and compared (b) by flow cytometry (FCM), gated on CD45^+^ CD4^+^ T cells according to IFN*γ* expression. (c, d) IFN*γ* staining (c) was performed to observe T_H_1 cell in the LP of the intestine and analyzed (d) according to pathological staining. (e–g) OT-II naïve T cells were cocultured with LPDCs from the LP of the small intestine sorted ex vivo in the presence of OVA323-339 and flagellin for 4 days before FCM analysis of T_H_1 (e, f). T cells from the coculture system were also sorted and succumb to immunoblotting (g); GAPDH was used as the loading control. And semiquantification of IFN*γ* level was calculated (g). (h, i) The level of RALDH was used to measure (h) and analyze (i) the ability of LPDCs to synthesize RA. (j, k) RA receptor inhibitor LE540 was added into the ex vivo coculture system consisting of naïve T cells and LPDCs (j), and T_H_1 differentiation was analyzed again (k). Data are expressed as the mean ± SD and represent three independent experiments involving 8 animals per group, *n* = 3 samples/group for western blotting. All significant *p* values are marked, and *p* < 0.05 is considered significant by the one-way ANOVA test.

To better understand local immune changes, we analyzed T cell subgroups in the LP of the small intestine (Figure S4). Interestingly, FCM analysis revealed that T_H_1 cells were relatively abundant in the LP, and importantly, TLR5 deficiency greatly reduced the abundance of T_H_1 (Figures 4(a) and 4(b)). Moreover, in WT mice, the abundance of T_H_1 cells decreased sharply after THS, whereas in the *Tlr5^−/−^* mice, the abundance remained low and seemingly unaffected by THS. IFN*γ* staining also confirmed this phenomenon (Figures 4(c) and 4(d)).

Ex vivo coculturing of LPDCs with naïve T cells exhibited T_H_1 differentiation changes similar to those described above (Figures 4(e) and 4(f)). In the sham group, the level of T_H_1 differentiation induced by LPDCs from WT mice was much higher than that from *Tlr5^−/−^* mice (*p* < 0.001). The ability of LPDCs to polarize T_H_1 in WT mice decreased significantly 12 (*p* = 0.0067) and 48 (*p* < 0.001) hours after THS, achieving higher levels of differentiation at 12 hours (*p* < 0.0001), thus indicating that this ability is time-dependent. Consistent with in situ observations, all the differences and time-dependent changes in T_H_1 differentiation disappeared in the absence of TLR5. Furthermore, the level of T_H_1 differentiation induced by LPDCs in WT mice was significantly higher 12 hours after THS compared with those in *Tlr5^−/−^* mice (*p* < 0.001). However, opposite trends were observed 48 hours after THS (*p* = 0.0203). In addition, in the absence of flagellin stimulation, significantly more T_H_1 differentiation was induced by the TLR5^−/−^ LPDCs 12 (*p* = 0.001) and 48 (*p* = 0.0231) hours after THS. Altogether, these results demonstrated that TLR5 is crucial for flagellin-stimulated LPDCs to regulate the homeostasis of T_H_1 cells in the small intestine. THS led to decreased LPDC function, which was restored to some extent by *Tlr5* knockout. The above results were confirmed by immunoblotting analysis of T cells from the ex vivo coculture system (Figure 4(g)).

#### 3.4.3. RA Released by TLR5^+^ LPDCs Played a Key Role in Modulating T_H_1 Polarization

RA release was quantified by RALDH expression. As seen in Figures 4(h) and 4(i), reduced RA production by WT-LPDCs was observed 48 hours after THS (*p* < 0.001). *Tlr5* knockout abolished RA production by LPDCs, regardless of whether the mice were subjected to THS or not. Furthermore, *Tlr5^−/−^* LPDCs did not synthesize RA in response to flagellin stimulation.

To determine whether RA controlled flagellin-activated LPDC regulation of T_H_1 polarization, we added the RA receptor inhibitor LE540 to the ex vivo coculture system of naïve T cells and LPDCs (Figure 4(j)). LE540 abolished T_H_1 differentiation induced by flagellin-activated LPDCs sorted from WT sham or post-THS mice. The percentages of T_H_1 differentiation decreased to a level equivalent to that at 48 hours after THS (Figure 4(k)). Moreover, LE540 had no impact on the ability of Tlr5^−/−^ LPDCs to polarize T_H_1. Therefore, RA synthesis that characterized LPDCs enabled them to polarize T_H_1 cells, thus suggesting that flagellin-TLR5-RA plays a critical role in inducing T_H_1 polarization by LPDCs in WT mice. Given the above findings, THS can severely impair the ability of TLR5^+^ LPDCs to synthesize RA and polarize T_H_1 in the LP of the small intestine. This rationale may partially explain the higher level of bacterial translocation. When *Tlr5* is knocked out, the LPDCs' ability to polarize T_H_1 decreases; however, this function seems more stable during THS.

### 3.5. RA Gavage Alleviated Bacterial Translocation in THS-Treated WT Mice

**Figure 5:**
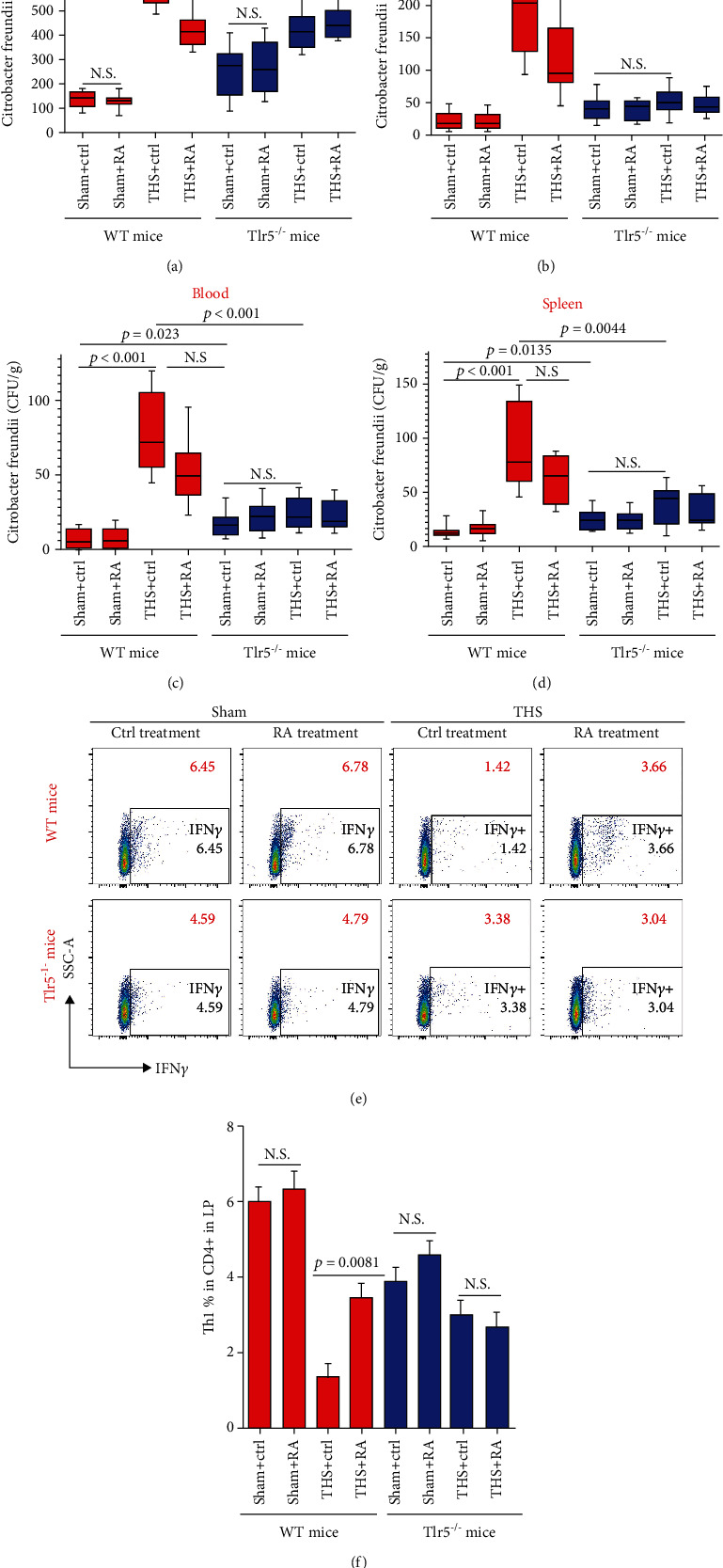
Effect of RA gavage on bacterial translocation in WT mice subjected to THS. Mice with or without RA treatment were sacrificed approximately 5 hours after *C. rodentium* challenge. Tissue samples were homogenized and cultured. Subsequently, bacterial abundance was calculated based on IVIS data. Samples were obtained from the MLNs (a), liver (b), blood (c), and spleen (d). Meanwhile, we determined the percentage of T_H_1 cells 48 hours after THS in the LP after RA treatment (e, f). Bacterial translocation data are expressed as median ± interquartile range. T_H_1 data are expressed as the mean ± SD, and each group contained 8 animals. All significant *p* values are marked, and *p* < 0.05 is considered significant by the one-way ANOVA test. Data represent three independent experiments.

To investigate the effects of RA treatment on bacterial translocation, we orally gavaged mice with RA and found that after THS in WT mice, RA attenuated bacterial translocation to the MLNs (Figure 5(a), *p* < 0.001), liver (Figure 5(b), *p* = 0.0258), and, to lesser extents, portal vein blood (Figure 5(c), *p* = 0.0673) and spleen (Figure 5(d), *p* = 0.1087). In contrast, bacterial translocation in *Tlr5^−/−^* mice after THS was not alleviated by RA (Figures 5(a)–5(d)). Interestingly, when we analyzed T_H_1 profiles in the LP after RA treatment, we found that RA increased the percentage of T_H_1 cells in WT mice after THS (Figures 5(e) and 5(f), *p* < 0.01). However, this was not observed in *Tlr5^−/−^* mice, thus suggesting that RA may be used as a therapeutic method to protect against bacterial translocation after THS.

### 3.6. TLR5 Deficiency Played a Positive Role in Alleviating the Impact of THS on Mouse Survival: RA Is a Potential Therapeutic Approach

WT mice treated with both THS and *C. rodentium* oral gavage survived for shorter periods than the sham-operated mice alone (*p* < 0.001), sham-operated mice administered with *C. rodentium* (*p* < 0.001), and mice subjected to THS alone (*p* = 0.0033) (Figure S5A). *Tlr5^−/−^* mice subjected to THS and *C. rodentium* oral gavage survived longer (*p* = 0.0352) than the correspondingly treated WT mice (Figure S5B). Moreover, the survival time of *Tlr5^−/−^* mice that experienced both THS and *C. rodentium* gavage was similar to that of mice treated with sham operations plus *C. rodentium* administration and mice treated with THS alone (Figure S5B). Therefore, TLR5 deficiency played a positive role in alleviating the impact of THS on mouse survival.

We then investigated the effect of RA on mouse survival after THS. To determine if RA could relieve bacterial translocation in WT mice after THS, we administered RA enterally and measured mouse survival. Although none of the groups treated with RA experienced significant improvement in their survival (Figures S5C and S5D), a similar trend was found between WT and *Tlr5^−/−^* mice after THS.

## 4. Discussion

Systemic infection after THS is associated with the dissemination of certain strains of bacteria that originate from the host gut and may become pathogenic following translocation. In this study, we demonstrated that THS increased gut mechanical permeability and upregulated RegIII*γ*-mediated bactericidal activity against G^+^ anaerobes, which subsequently shaped the composition of the microbiota, and further compromised the LPDC-mediated TLR5-RA pathway that polarized T_H_1 differentiation. These processes ultimately resulted in the translocation and dissemination of commensal bacteria. More importantly, our experiments provided sufficient evidence supporting the contribution of the TLR5 pathway to post-THS infection. It is widely acknowledged that intestinal mechanical barrier damage, gut flora dysbiosis, and compromised mucosal immunity are the three most classic and pivotal factors promoting bacterial translocation [38], and in the present study, we found that TLR5 deficiency influenced the latter two. It should be noted that our findings related to changes in the composition of the gut microbiota through time and observations of bacterial translocation implied selectivity in barrier leakage, survival, or expansion of bacteria because there were significant differences between WT and *Tlr5^−/−^* mice. Our data suggested that *Tlr5* knockout plays a critical role in alleviating bacterial translocation.

Contrasts between the gut microbiota of WT and *Tlr5^−/−^* mice have been studied previously and showed that neonatal TLR5 expression strongly influenced the composition of the microbiota throughout life [39]. Here, we found that the levels of *Proteobacteria* increased, whereas those of the *Bacteroidetes* decreased 12 hours after THS. These observations were similar to those seen in colitic *Tlr5^−/−^* and WT mice as reported in another study [40]. At the genus level, there was a marked decrease in the abundance of G^+^ anaerobes, including *Turicibacter*, *Allobaculum*, and *Clostridiaceae* in WT mice after THS, whereas no significant changes occurred in *Tlr5^−/−^* mice. Anaerobic bacteria are the main component of the gut biological barrier and play a vital role in its function. Decreasing the levels of anaerobic bacteria can result in a weakened biological barrier, which can in turn facilitate bacterial translocation.

Moreover, the alpha diversity index values describing the gut microbiota in *Tlr5^−/−^* mice were higher than those in WT mice, which meant that the enteric microbiota environment of *Tlr5^−/−^* mice is more stable and less influenced by THS. To our dismay, the UniFrac distance between the gut communities of WT and *Tlr5^−/−^* mice was not close, even though the mice were housed together from birth. This observation might be explained by the specific selection mechanism imposed by TLR5 that, although is restricted to the early postnatal period, may exert an effect that lasts until adulthood. However, environmental factors may also exert a strong and possibly dominant effect during the early period [41].

Importantly, a significant increase in intestinal RegIII*γ* expression was documented in WT mice after THS. Recent studies have demonstrated that intestinal microbiota plays a critical role in regulating innate mucosal immunity, which in turn affects maintenance of host-microbiota mutualism [42]. Antimicrobial proteins (AMPs) produced by the intestinal epithelium are effector molecules that provide the first line of defense against invading commensal and pathogenic organisms. The AMP RegIII*γ* is a secreted C-type lectin with bactericidal activity against G^+^ bacteria including G^+^ anaerobes and is induced by intestinal commensal bacteria [20]. In the present study, we found that TLR5 was necessarily and intensely required for the activation of RegIII*γ* synthesis. Intriguingly, RegIII*γ* synthesis was nearly completely suppressed after *Tlr5* knockout, even after flagellin stimulation. Moreover, after THS, the RegIII*γ* expression in intestinal epithelial and Paneth cells along the length of the small intestine was induced after exposure to bacterial flagellin. The low microcirculation induced by THS increased susceptibility of the intestinal lining and the likelihood of interactions between flagellated bacteria and subepithelial immune cells. It has been reported that flagellin-mediated RegIII*γ* could not be directly upregulated and required TLR5 activation in hematopoietic cells [20]. However, we found that RegIII*γ* synthesis at 48 hours was lower than that at 12 hours. We hypothesized that 48 hours after THS, the subepithelial immune cells might have been severely injured, which impaired upregulation of flagellin-mediated RegIII*γ* production, although contact with bacterial flagellin was potentially increased.

Consistent with its involvement in regulating intestinal microbiota homeostasis, the TLR5 pathway played a crucial role in mucosal immunity in our study. THS severely impaired the ability of LPDCs to synthesize RA and polarize T_H_1 in the LP of the small intestine, which might also explain the higher rate of bacterial translocation and poorer survival of WT mice. The TLR5-RA signaling pathway played a key role in this process. As previously confirmed, T_H_1 responses were induced by intestinal bacterial flagellin via modulation of the TLR5-RA signaling pathway in LPDCs. As a signaling molecule for APCs, RA triggers the T_H_1 immune response, characterized by the secretion of INF-*γ*, IL-2, and lymphotoxin-*α*, which are very important for resistance to intracellular pathogens including viruses, bacteria, protozoa, and fungi [21]. Interestingly, *Tlr5* knockout resulted in highly impaired synthesis of RA by LPDCs, which was not influenced by THS. This observation provided strong evidence that *Tlr5* knockout exerts protective effects against bacterial translocation after THS. We hypothesized that a compensatory mechanism for TLR5-RA-independent T_H_1 polarization is developed during embryonic development in the *Tlr5* knockout mice, and THS can barely influence this compensatory pathway. Mice expressing this pathway maintain their ability to induce T_H_1 differentiation in the LP and defend against bacterial translocation.

Notwithstanding that RA has been reported to have therapeutic effects by protecting intestinal mucosa and alleviating bowel inflammation, its function in bacterial translocation is currently poorly understood [43–45]. Consistent with its protective role, enteral RA supplementation not only facilitated T_H_1 differentiation in the LP but also diminished bacterial translocation in WT mice. However, RA supplementation did not affect *Tlr5^−/−^* mice after THS, thus emphasizing the physiological relevance of the TLR5-RA pathway. Although limited impact of RA on survival was observed, we could not ignore the significance of RA, and some factors might have concealed its true effects. Nonetheless, our findings suggested that RA treatment was inclined to improve mouse survival after THS. However, the number of mice in each group was not large enough, which could have limited our study findings. Moreover, RA exhibited a potent ability to block bacterial translocation to MLNs and liver but a relatively weaker ability to alleviate bacterial translocation to the systemic circulation, including the portal vein blood and spleen. Accordingly, the influence of RA effects on mice's survival mostly depends on its effectiveness in blocking bacterial translocation to systemic circulation; after all, compared to the quantity of translocated bacteria, whether bacteria translocate accounts more for the lethal systemic bacteremia. Furthermore, changes in the gut microbiota and immunological alterations in the limited time frame after THS determine whether bacterial translocation will occur and the number of bacteria translocated. Systemic infection resulting from bacterial translocation to the circulation is the main cause of mortality. Although bacterial translocation is a transient process, bacterial translocation-induced local and systemic infection is a long story, including bacterial amplification after translocation, further spread, and interactions with the innate immunity, which determines whether the infection is controlled. In a word, we cannot deny RA's important value on improving the intestinal mucosal immunity and blocking bacterial translocation; accordingly, modulating the time/duration of RA treatment could have huge prospects as a prophylactic approach against bacterial translocation. Moreover, our findings also highlighted the need to investigate gut microbiota interventions, such as probiotics and fecal transplants, which have been documented to have superior efficacy, although they are considered optional therapeutic approaches [46, 47].

In summary, we have demonstrated that TLR5 signaling is pivotal in regulating RegIII*γ* expression and RA-mediated T_H_1 differentiation by LPDCs. These processes contribute to maintaining gut microbiota homeostasis and mucosal immunity and eventually defend against intestinal bacterial translocation and enterogenous infection after THS. We hypothesized the existence of stable TLR5-independent mechanisms that are relatively unaffected by THS and thus explain the relatively stable composition of gut microbiota and LPDC-induced polarization of T_H_1 cells in *Tlr5^−/−^* mice. Whether LPDCs or gut microbiota are potential therapeutic targets to prevent bacterial translocation after THS warrants further investigation.

However, there are some limitations in our study. The mechanisms underlying the decreased ability of LPDCs to polarize T_H_1 after THS are still unknown. It also remains unclear whether these mechanisms indirectly upregulate RegIII*γ* in hematopoietic cell lines.

More importantly, *Tlr5* knockout seemed to restore the function of impaired LPDCs after THS; however, the underlying mechanisms were not explored in our study. Although most TLR5^+^ DCs gather in the intestinal lamina propria, general knockout of *Tlr5* unavoidably affects the whole body immune system and gut microbiota during the development process. Most importantly, the results of RegIII*γ*, RA secretion, T_H_1 cell differentiation, commensal bacterial translocation, and survival demonstrated the outcomes associated with TLR5 deficiency. Our findings corroborated that TLR5 plays an important role in intestinal mucosal immunity, bacterial translocation, and enterogenous infection. Accordingly, this study provided the basis for further studies aiming at uncovering details of the TLR5 pathway and its downstream signals. A study by Friedrich et al. [48] reported that during intestinal infection, functional MyD88 signaling (downstream signal of TLR5) in CD11c^+^ cells was sufficient to activate intestinal DCs, induce T_H_ cell development, generate enhanced epithelial barrier integrity, and increase expression of the antimicrobial peptide RegIII*γ* by epithelial cells, which was consistent with our findings. In contrast, restricting MyD88 signaling to several other cell types, including macrophages, T cells, or ILC3, did not induce efficient intestinal immune responses upon infection. Moreover, *Tlr5^f/f^*, *CD11c-cre*, *Tlr5^f/f^*, and *Vil1-cre* mice are urgently wanted for more accurate researches of TLR5's roles in DCs and intestinal epithelia. Furthermore, inducible *Tlr5* knockout is also an ideal try to favor TLR5 research in the special time and space. Finally, bone marrow transplant and clodronate experiments also deserve implementation to verify the important value of TLR5^+^ DCs.

## Data Availability

Data are available from corresponding author Ting-Bo Liang (liangtingbo@zju.edu.cn) or first author Yun Zhang (bigzyun1977@zju.edu.cn).

## Supplementary Materials

Supplementary Materials Supplementary Figures S1–S5 and supplementary figure legends. Figure S1: bacterial translocation model. Figure S2: effect of TLR5 deficiency on the profile of microbial composition after THS. Figure S3: identification and basic characteristics of TLR5^+^ LPDCs. Figure S4: flowchart of T_H_1 analysis in the LP. Figure S5: survival analysis.

## References

[1] Kimura F., Shimizu H., Yoshidome H., Ohtsuka M., Miyazaki M. (2010). Immunosuppression following surgical and traumatic injury. *Surgery Today*.

[2] Singh V., Roth S., Llovera G. (2016). Microbiota dysbiosis controls the neuroinflammatory response after stroke. *The Journal of Neuroscience*.

[3] Stanley D., Mason L. J., Mackin K. E. (2016). Translocation and dissemination of commensal bacteria in post-stroke infection. *Nature Medicine*.

[4] Xiao L., Feng Q., Liang S. (2015). A catalog of the mouse gut metagenome. *Nature Biotechnology*.

[5] Sartor R. B. (2008). Microbial influences in inflammatory bowel diseases. *Gastroenterology*.

[6] Benakis C., Brea D., Caballero S. (2016). Commensal microbiota affects ischemic stroke outcome by regulating intestinal *γδ* T cells. *Nature Medicine*.

[7] Uematsu S., Jang M. H., Chevrier N. (2006). Detection of pathogenic intestinal bacteria by toll-like receptor 5 on intestinal CD11c^+^ lamina propria cells. *Nature Immunology*.

[8] Uematsu S., Fujimoto K., Jang M. H. (2008). Regulation of humoral and cellular gut immunity by lamina propria dendritic cells expressing Toll-like receptor 5. *Nature Immunology*.

[9] Uematsu S., Akira S. (2009). Immune responses of TLR5(+) lamina propria dendritic cells in enterobacterial infection. *Journal of Gastroenterology*.

[10] Fujimoto K., Karuppuchamy T., Takemura N. (2011). A new subset of CD103+CD8alpha+ dendritic cells in the small intestine expresses TLR3, TLR7, and TLR9 and induces Th1 response and CTL activity. *Journal of Immunology*.

[11] Kinnebrew M. A., Buffie C. G., Diehl G. E. (2012). Interleukin 23 production by intestinal CD103^+^CD11b^+^ dendritic cells in response to bacterial flagellin enhances mucosal innate immune defense. *Immunity*.

[12] Wang Q., Liu C., Zhu F. (2010). Reoxygenation of hypoxia-differentiated dentritic cells induces Th1 and Th17 cell differentiation. *Molecular Immunology*.

[13] Brandl K., Plitas G., Mihu C. N. (2008). Vancomycin-resistant enterococci exploit antibiotic-induced innate immune deficits. *Nature*.

[14] Nowarski R., Jackson R., Flavell R. A. (2017). The stromal intervention: regulation of immunity and inflammation at the epithelial-mesenchymal barrier. *Cell*.

[15] Palomo J., Dietrich D., Martin P., Palmer G., Gabay C. (2015). The interleukin (IL)-1 cytokine family - balance between agonists and antagonists in inflammatory diseases. *Cytokine*.

[16] Zheng Y., Valdez P. A., Danilenko D. M. (2008). Interleukin-22 mediates early host defense against attaching and effacing bacterial pathogens. *Nature Medicine*.

[17] Thaiss C. A., Levy M., Suez J., Elinav E. (2014). The interplay between the innate immune system and the microbiota. *Current Opinion in Immunology*.

[18] Chassaing B., Ley R. E., Gewirtz A. T. (2014). Intestinal epithelial cell toll-like receptor 5 regulates the intestinal microbiota to prevent low-grade inflammation and metabolic syndrome in mice. *Gastroenterology*.

[19] Oh J. Z., Ravindran R., Chassaing B. (2014). TLR5-mediated sensing of gut microbiota is necessary for antibody responses to seasonal influenza vaccination. *Immunity*.

[20] Kinnebrew M. A., Ubeda C., Zenewicz L. A., Smith N., Flavell R. A., Pamer E. G. (2010). Bacterial flagellin stimulates Toll-like receptor 5-dependent defense against vancomycin-resistant Enterococcus infection. *The Journal of Infectious Diseases*.

[21] Mizubuchi H., Yajima T., Aoi N., Tomita T., Yoshikai Y. (2005). Isomalto-oligosaccharides polarize Th1-like responses in intestinal and systemic immunity in mice. *The Journal of Nutrition*.

[22] Yu W., du H., Fu Q., Cui N., du C. (2014). The influence of Th1/Th2 and CD4^+^ regulatory t cells of mesenteric lymph nodes on systemic lipopolysaccharide. *Polish Journal of Pathology*.

[23] Mahapatro M., Erkert L., Becker C. (2021). Cytokine-mediated crosstalk between immune cells and epithelial cells in the gut. *Cell*.

[24] Tang Y., Xia X. F., Zhang Y. (2012). Establishment of an experimental mouse model of trauma-hemorrhagic shock. *Experimental Animals*.

[25] Wiles S., Clare S., Harker J. (2004). Organ specificity, colonization and clearance dynamics in vivo following oral challenges with the murine pathogen Citrobacter rodentium. *Cellular Microbiology*.

[26] Wiles S., Dougan G., Frankel G. (2005). Emergence of a ‘hyperinfectious’ bacterial state after passage of Citrobacter rodentium through the host gastrointestinal tract. *Cellular Microbiology*.

[27] Zhang Y., Zhang J., Xu T. (2017). Allicin ameliorates intraintestinal bacterial translocation after trauma/hemorrhagic shock in rats: the role of mesenteric lymph node dendritic cell. *Surgery*.

[28] Manichanh C., Rigottier-Gois L., Bonnaud E. (2006). Reduced diversity of faecal microbiota in Crohn's disease revealed by a metagenomic approach. *Gut*.

[29] Fadrosh D. W., Ma B., Gajer P. (2014). An improved dual-indexing approach for multiplexed 16S rRNA gene sequencing on the Illumina MiSeq platform. *Microbiome*.

[30] Jervis-Bardy J., Leong L. E., Marri S. (2015). Deriving accurate microbiota profiles from human samples with low bacterial content through post-sequencing processing of Illumina MiSeq data. *Microbiome*.

[31] Asakura M., Kitakaze M., Takashima S. (2002). Cardiac hypertrophy is inhibited by antagonism of ADAM12 processing of HB- EGF: metalloproteinase inhibitors as a new therapy. *Nature Medicine*.

[32] Yoshida J., Yamamoto K., Mano T. (2004). AT1 receptor blocker added to ACE inhibitor provides benefits at advanced stage of hypertensive diastolic heart failure. *Hypertension*.

[33] Atarashi K., Nishimura J., Shima T. (2008). ATP drives lamina propria T_H_17 cell differentiation. *Nature*.

[34] Klemann C., Raveney B. J., Klemann A. K. (2009). Synthetic retinoid AM80 inhibits Th17 cells and ameliorates experimental autoimmune encephalomyelitis. *The American Journal of Pathology*.

[35] Zhang J., Yu W. Q., Wei T. (2020). Effects of short-peptide-based enteral nutrition on the intestinal microcirculation and mucosal barrier in mice with severe acute pancreatitis. *Molecular Nutrition & Food Research*.

[36] Lu Q., Xu D. Z., Sharpe S. (2011). The anatomic sites of disruption of the mucus layer directly correlate with areas of trauma/hemorrhagic shock-induced gut injury. *The Journal of Trauma*.

[37] Harrois A., Baudry N., Huet O. (2013). Synergistic deleterious effect of hypoxemia and hypovolemia on microcirculation in intestinal villi. *Critical Care Medicine*.

[38] Perez-Lopez A., Behnsen J., Nuccio S. P., Raffatellu M. (2016). Mucosal immunity to pathogenic intestinal bacteria. *Nature Reviews. Immunology*.

[39] Fulde M., Sommer F., Chassaing B. (2018). Neonatal selection by Toll-like receptor 5 influences long-term gut microbiota composition. *Nature*.

[40] Carvalho F. A., Koren O., Goodrich J. K. (2012). Transient inability to manage proteobacteria promotes chronic gut inflammation in TLR5-deficient mice. *Cell Host & Microbe*.

[41] Ubeda C., Lipuma L., Gobourne A. (2012). Familial transmission rather than defective innate immunity shapes the distinct intestinal microbiota of TLR-deficient mice. *The Journal of Experimental Medicine*.

[42] Martens E. C., Neumann M., Desai M. S. (2018). Interactions of commensal and pathogenic microorganisms with the intestinal mucosal barrier. *Nature Reviews Microbiology*.

[43] Frey-Wagner I., Fischbeck A., Cee A. (2013). Effects of retinoids in mouse models of colitis: benefit or danger to the gastrointestinal tract?. *Inflammatory Bowel Diseases*.

[44] Mielke L. A., Jones S. A., Raverdeau M. (2013). Retinoic acid expression associates with enhanced IL-22 production by *γδ* T cells and innate lymphoid cells and attenuation of intestinal inflammation. *The Journal of Experimental Medicine*.

[45] Ozdemir R., Yurttutan S., Sari F. N. (2013). All-trans-retinoic acid attenuates intestinal injury in a neonatal rat model of necrotizing enterocolitis. *Neonatology*.

[46] Shanahan F., Quigley E. M. (2014). Manipulation of the microbiota for treatment of IBS and IBD--challenges and controversies. *Gastroenterology*.

[47] Sartor R. B., Wu G. D. (2017). Roles for intestinal bacteria, viruses, and fungi in pathogenesis of inflammatory bowel diseases and therapeutic approaches. *Gastroenterology*.

[48] Friedrich C., Mamareli P., Thiemann S. (2017). MyD88 signaling in dendritic cells and the intestinal epithelium controls immunity against intestinal infection with C. rodentium. *PLoS Pathogens*.

