# Supplementary material for: Role of TLR5 in the Translocation and Dissemination of Commensal Bacteria in the Intestine after Traumatic Hemorrhagic Shock
Source: J Immunol Res. 2021 Nov 19;2021:6417658. doi: 10.1155/2021/6417658 (PMC8626180; doi:10.1155/2021/6417658)
Supplement: Supplementary Materials — Supplementary Figures S1–S5 and supplementary figure legends. Figure S1: bacterial translocation model. Figure S2: effect of TLR5 deficiency on the profile of microbial composition after THS. Figure S3: identification and basic characteristics of TLR5+ LPDCs. Figure S4: flowchart of TH1 analysis in the LP. Figure S5: survival analysis. [file 6417658.f1.pdf]

SUPPLEMENTARY FIGURES AND FIGURE LEGENDS

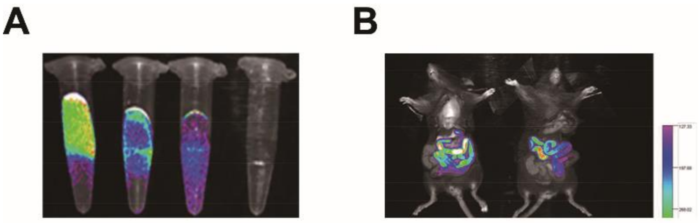

Fig. S1. Bacterial translocation model.

Bioluminescent *C. rodentium* was employed to monitor bacterial translocation. (A, B) Bioluminescent *C. rodentium* could be visualized using an in vivo imaging system (IVIS). (A) The bioluminescence intensity decreased as the bacterial suspension was serially diluted. (B) IVIS image of bacteria in the intestine of mice after oral gavage.

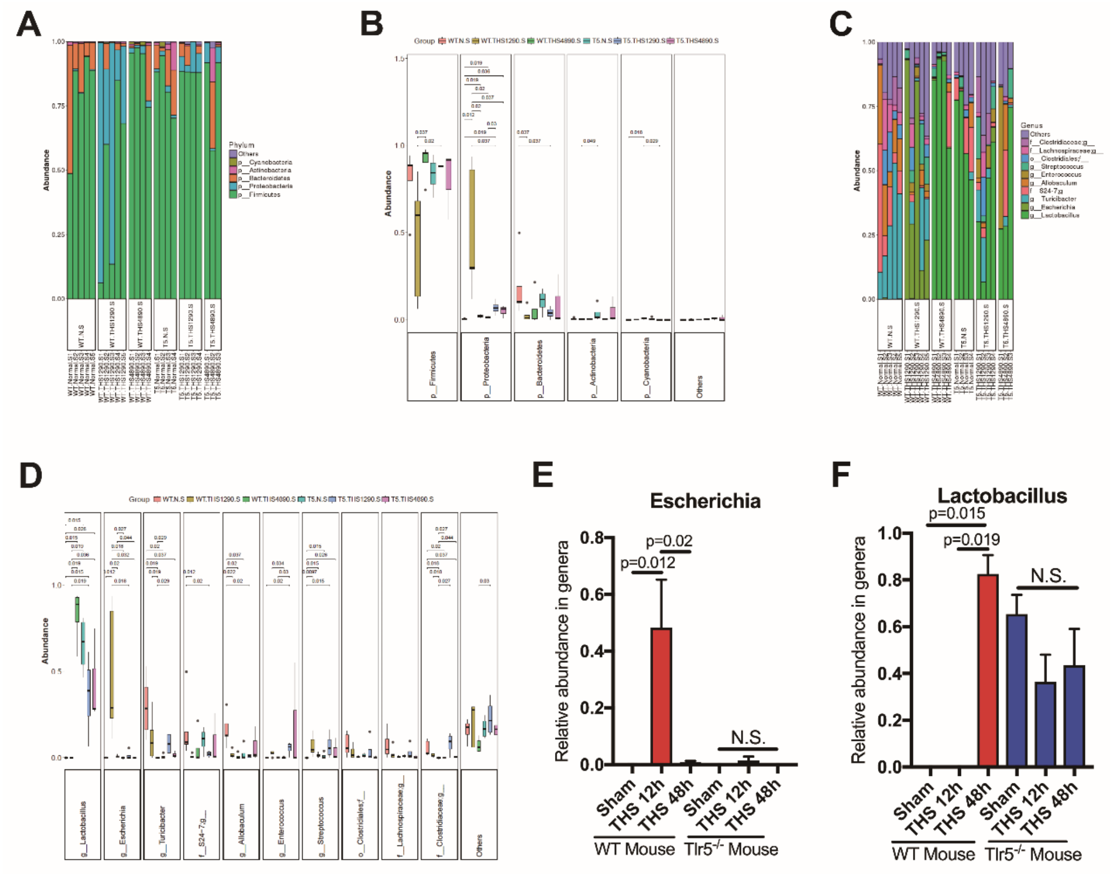

Fig. S2. Effect of TLR5 deficiency on the profile of microbial composition after THS

Microbial composition in the small intestines of sham-operated (WT.N.S (n=5) and T5.N.S (n=4)), 12 hours post-THS (WT.THS1290.S (n=5), T5.THS1290.S (n=4)) mice, 48 hours post-THS (WT.THS4890.S (n=4), T5.THS4890.S (n=3)) of different genotypes (WT and *Tlr5*$^{-/-}$) in the phylum level (A and B) and genera level (C and D). (E) Relative abundance changes of Escherichia genera (E) and Lactobacillus genera (F) within small intestine of sham-operated and post-THS WT or *Tlr5*$^{-/-}$ mice. Data represent the mean ± SD, and number of mice in each group is described before. All significant p values are indicated, $p<0.05$ is considered significant by the one-way ANOVA test.

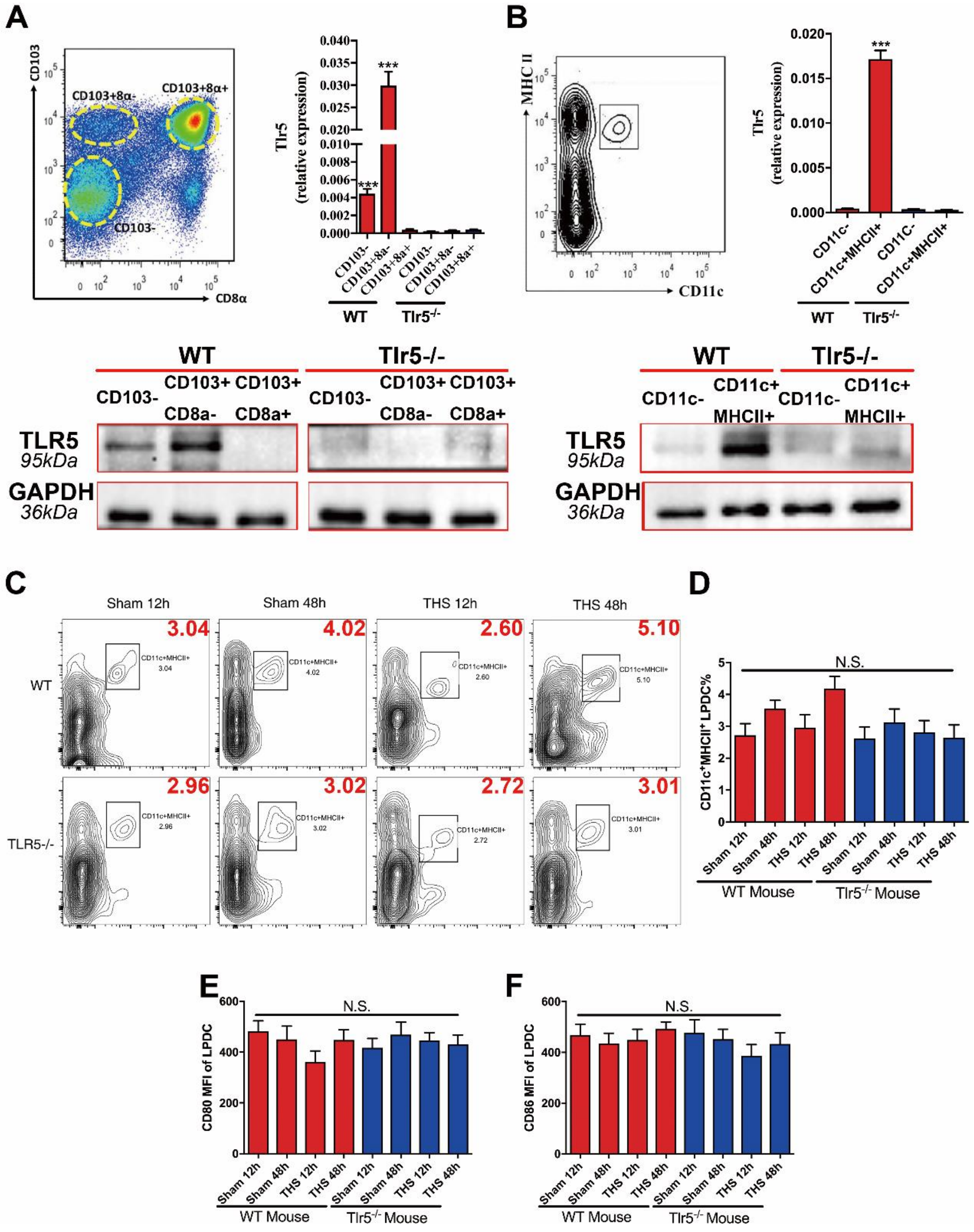

A
CD103
CD103+8α-
CD103+8α+
CD103-
CD8α
Tlr5
(relative expression)
***
***
CD103-
CD103+8a-
CD103+8a+
WT
Tlr5-/-
B
MHC II
CD11c
Tlr5
(relative expression)
***
CD11c-
CD11c+MHCII+
WT
Tlr5-/-
WT
Tlr5-/-
CD103-
CD103+ CD8a-
CD103+ CD8a+
TLR5
95kDa
GAPDH
36kDa
CD11c-
CD11c+ MHCII+
C
Sham 12h
Sham 48h
THS 12h
THS 48h
WT
TLR5-/-
3.04
4.02
2.60
5.10
2.96
3.02
2.72
3.01
CD11c+MHCII+
D
N.S.
CD11c+MHCII+ LPDC%
Sham 12h
Sham 48h
THS 12h
THS 48h
WT Mouse
Tlr5-/- Mouse
E
N.S.
CD80 MFI of LPDC
F
N.S.
CD86 MFI of LPDC

Fig. S3. Identification and basic characteristics of TLR5$^+$ LPDCs

CD103 plus CD8a (A) or CD11c plus MHCII (B) were used to sort the different subgroups of LPDCs from the intestinal LP, and TLR5 expression was analyzed by RT-PCR and western blot. The percentages of CD11c$^+$ MHCII$^+$ LPDCs in the LP among the groups were analyzed (C) and compared (D). (E) CD80 (E) and CD86(F) expression in the CD11c$^+$MHCII$^+$ LPDCs from each group were compared. Each group contained 8 animals, and data are expressed as the mean ± SD, representative of three independent experiments. All significant p values are marked and $p<0.05$ is considered significant by the one-way ANOVA test.

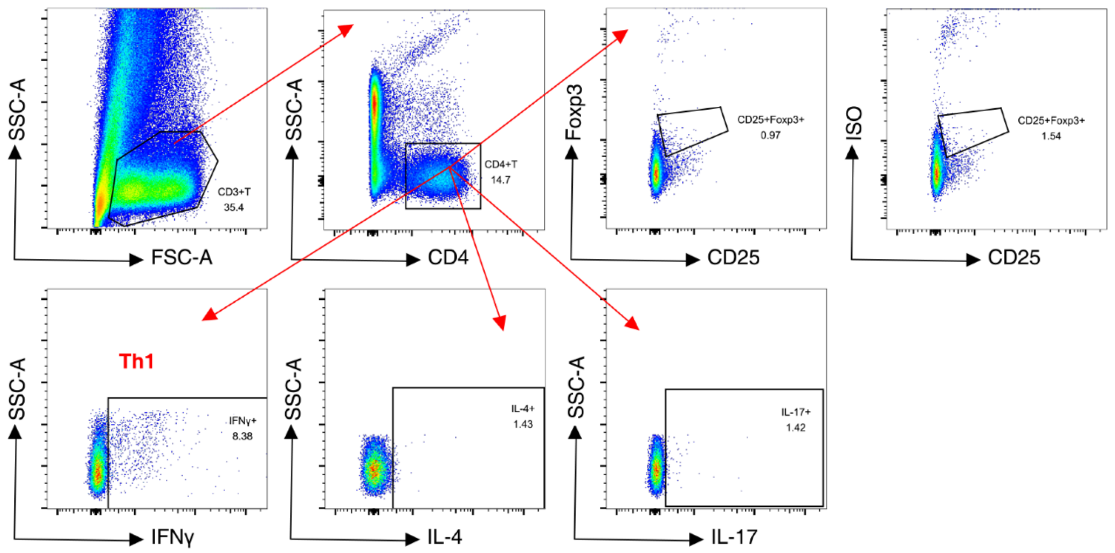

Fig. S4. Flowchart of Th1 analysis in the LP

T cell subgroups were gated on CD4+ cells from LP single cells. Then Th1 cells were gated on IFNγ^{+}, Th2 cells were gated on IL-4^{+}, Th17 cells were gated on IL-17^{+}, and Treg cells were gated on CD25^{+}Foxp3^{+}.

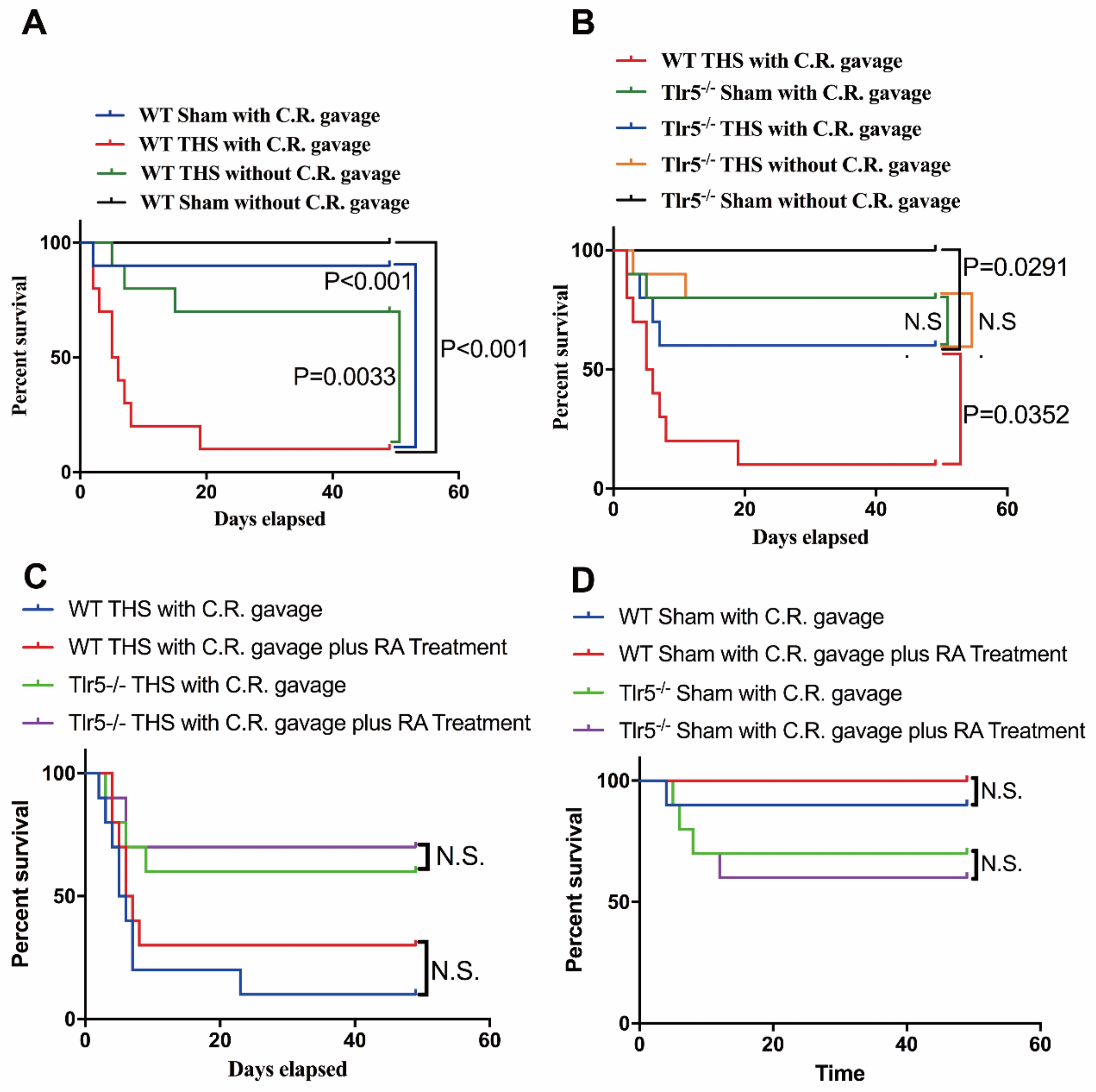

Fig. S5. Survival analysis.

Mouse survival was monitored for 2 months every 2 or 3 days after the treatments. Comparisons were made between the different groups shown in the figure (A–D). Each group contained 10 animals. The data represent three independent experiments. Survival was analyzed using the Kaplan–Meier method with log-rank test, and $p<0.05$ is considered significant.
